# Effects of NAC and Gallic Acid on the Proliferation Inhibition and Induced Death of Lung Cancer Cells with Different Antioxidant Capacities

**DOI:** 10.3390/molecules27010075

**Published:** 2021-12-23

**Authors:** Chen-Yi Liao, Ting-Chien Wu, Shun-Fa Yang, Jinghua Tsai Chang

**Affiliations:** 1 Institute of Medicine, Chung Shan Medical University, 110 Sec. 1, Chien-Kuo N. Road, Taichung 40203, Taiwan; firmsky02@yahoo.com.tw (C.-Y.L.); s0903014@gm.csmu.edu.tw (T.-C.W.); ysf@csmu.edu.tw (S.-F.Y.); 2 CSMU Lung Cancer Research Center, Chung Shan Medical University, 110 Sec. 1, Chien-Kuo N. Road, Taichung 40203, Taiwan; 3 Divisions of Medical Oncology and Pulmonary Medicine, Chung Shan Medical University Hospital, 110 Sec. 1, Chien-Kuo N. Road, Taichung 40203, Taiwan

**Keywords:** *PERP-428* SNP, *N*-acetylcysteine, gallic acid, oxidative stress, antioxidant, pro-oxidant, cell survival

## Abstract

*N*-acetylcysteine (NAC) is a recognized antioxidant in culture studies and treatments for oxidative stress-related diseases, but in some cases, NAC is a pro-oxidant. To study the effect of NAC on cell proliferation in the presence or absence of ROS stress, we used the stable ROS generator gallic acid (GA) to treat CL1-0 lung cancer cell models with different antioxidant activities. Different antioxidant activities were achieved through the ectopic expression of different *PERP-428* single nucleotide polymorphisms. GA increased ROS levels in CL1-0/*PERP-428C* cells and caused cell death but had no effect on CL1-0/*PERP-428G* cells within 24 h. We found that 0.1 mM NAC eliminated GA-induced growth inhibition, but 0.5 mM NAC enhanced GA-induced CL1-0/*PERP-428C* cell death. However, in the absence of GA, NAC exceeding 2 mM inhibited the growth of CL1-0/*PERP-428G* cells more significantly than that of CL1-0/*PERP-428C* cells. Without GA, NAC has an antioxidant effect. Under GA-induced ROS stress, NAC may have pro-oxidant effects. Each cell type has a unique range of ROS levels for survival. The levels of ROS in the cell determines the sensitivity of the cell to an antioxidant or pro-oxidant. Cells with different antioxidant capacities were used to show that the intracellular ROS level affects NAC function and provides valuable information for the adjuvant clinical application of NAC.

## 1. Introduction

**Figure 1:**
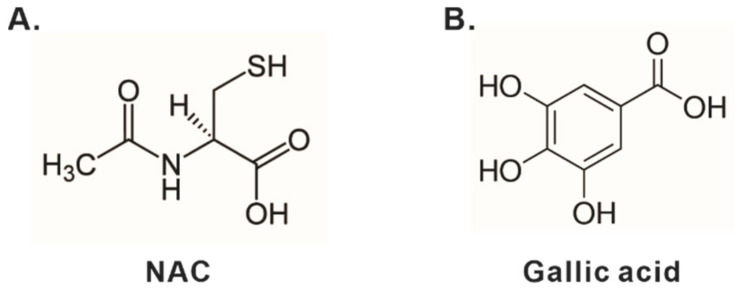
The structures of NAC (**A**) and gallic acid (**B**).

NAC is an acetylated variant of L-cysteine, a widely used antioxidant (Figure 1A). As a thiol compound, NAC can be used as a direct or an indirect antioxidant. NAC can directly react with H_2_O_2_ and O_2_^•−^, but its activity is negligible compared with that of catalase and superoxide dismutase. However, hypochlorous acid (HOCl) generated by activated neutrophils and monocytes is highly reactive to NAC [1]. L-cysteine is a building block of GSH and the rate-limiting substance for the synthesis of GSH through γ-GCS [1]. Since GSH is an antioxidant and an important cofactor for antioxidant enzymes that protects cells from free radical damage [2,3], a major part of the indirect antioxidant effect of NAC is conferred by GSH. A previous report showed that 2-cyclohexene-1-one (CHX) can cause short-term spatial memory deficits in rats and mice, and NAC can rescue GSH depletion and restore cognitive deficits [4]. In addition, the cysteine generated from NAC can be catabolized into reduced sulfur H_2_S and 3-mecapto-pyruvate (3MP) and then further converted into sulfane sulfur species in mitochondria, acting as scavengers for oxidants [5]. Clinically, NAC has been used to treat acetaminophen overdose [6] and as a mucolytic agent to reduce the viscosity of mucus by breaking disulfide bonds in high molecular weight glycoproteins [7].

Although NAC is a recognized antioxidant, it also has pro-oxidant effects that depend on the types of free radicals encountered. Studies have shown that NAC can function as an effective free radical scavenger against peroxyl free radicals (ROO^•^) and alkoxy radicals (RO^•^) generated by the decomposition of AAPH (2,2′-azobis-(2-amidinopropane) dihydrochloride) [8]. However, when the Fenton reaction of H_2_O_2_ produces hydroxyl radicals (HO^•^), NAC acts as a pro-oxidant and forms thiyl radicals (NAC^•^) [8].

The p53 apoptosis effector related to *PMP22* (*PERP*) encodes a tetraspan membrane protein comprising 193 amino acids. *PERP* is activated in p53-mediated apoptosis [9] and modulates apoptosis via a caspase-dependent pathway [10,11]. We previously identified *PERP-428* SNPs (single nucleotide polymorphisms) *PERP-428C* and *PERP-428G*, and found that the antioxidant activity of CL1-0 lung cancer cells expressing *PERP-428C* was lower than that of cells expressing *PERP-428G*. We used gallic acid (GA) (Figure 1B), a stable ROS generator, to study how *PERP-428* SNPs differentially regulate antioxidant activities and found that *PERP-428C* reduces the expression of the antioxidant enzymes catalase and glutathione reductase through the PTEN/MDM2/p53 pathway [12]. We unexpectedly found that 10 mM NAC enhanced GA-induced cell death. You and Park also found that 2 mM NAC enhanced the growth inhibition and death of human pulmonary fibroblasts treated with GA [13]. Since the antioxidant or pro-oxidant role of NAC is closely related to the level of H_2_O_2_ [8], we examined the effects of NAC on proliferation and cell death in the presence or absence of GA-induced ROS in CL1-0 cell models with different antioxidant activities. The different antioxidant activities of CL1-0 cells are achieved by the ectopic expression of *PERP-428* SNPs. We found that cells expressing *PERP-428G* (CL1-0/*PERP-428G*) with higher antioxidant activity were more sensitive to NAC than CL1-0/*PERP-428C* cells with lower antioxidant activity. On the other hand, cells with lower antioxidant capacity are more sensitive to NAC and GA cotreatment than cells with higher antioxidant activity. NAC has been used orally or intravenously in various clinical trials [14]. We hope this study provides information for NAC used in cell culture research and reminds researchers to assess the role of NAC in adjuvant therapy in every clinical application, especially when ROS levels are high.

## 2. Results

### 2.1. NAC Enhances the Growth-Inhibitory Effect of GA-Induced Lung Cancer Cells

**Figure 2:**
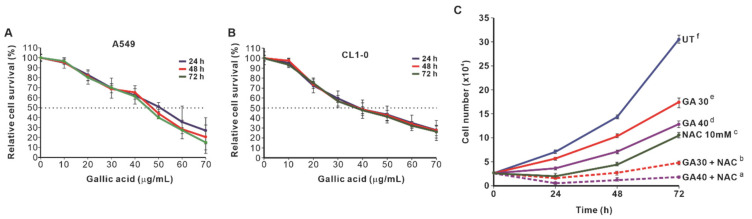
The effects of GA and NAC treatment on the inhibition of cell survival and proliferation of lung cancer cells. A549 cells (**A**) and CL1-0 cells (**B**) were treated with different concentrations of GA (0, 10, 20, 30, 40, 50, 60, 70 μg/mL) for 24, 48 and 72 h of treatment. The cell survival rate of a concentration of GA cultured for 24 h (SR_24,[GA]_) was calculated by dividing the number of cells in each GA treated group for 24 h (N_24,[GA]_) by the cell number of the untreated group for 24 h (N_24,[GA]=0_). The relative survival rate of the untreated group for each incubation time was defined as 100%. (**C**) NAC (10 mM) enhanced GA-induced inhibition of A549 cell proliferation. One-way ANOVA and the Scheffé test were used for analysis of NAC and NAC/GA treatment groups; F(5,12) = 1077.164, *p* = 1.76 × 10^−15^. The letters in the figure indicate the homogeneous subsets, and data sharing the same letter are not significantly different as determined by the Scheffé test (*p* > 0.05). GA30 (30 μg/mL), GA40 (40 μg/mL).

**Figure 3:**
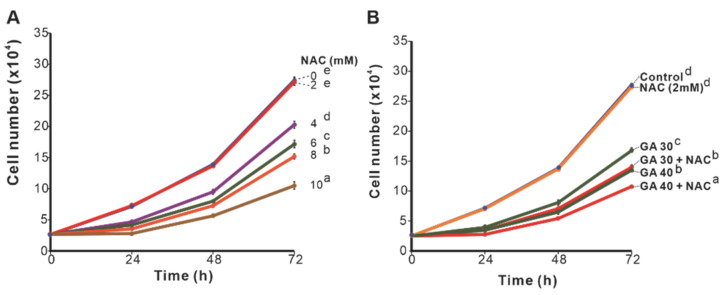
The effects of different concentrations of NAC on the proliferation of A549 cells. (**A**) NAC (2 mM) did not inhibit cell proliferation. However, the concentration of NAC was higher than 4 mM, which inhibited the proliferation of A549 cells in a dose-dependent manner. One-way ANOVA and the Scheffé test were used to analyze the effects of different concentrations of NAC on cell proliferation; F(5,12) = 506.427, *p* = 1.6 × 10^−13^. (**B**) NAC (2 mM) alone did not inhibit cell proliferation, but enhanced GA-induced inhibition of proliferation in A549 cells; GA30 (30 μg/mL), GA40 (40 μg/mL). One-way ANOVA and the Scheffé test were used for analysis of cell proliferation; F(5,12) = 1644.47, *p* = 1.4 × 10^−14^. The letters in the figure indicate the homogeneous subsets, and data sharing the same letter are not significantly different as determined by the Scheffé test (*p* > 0.05).

GA is a polyhydroxy phenolic compound present in various plants and fruits. This molecule acts as an antioxidant at low concentrations, but at high concentrations, it acts as an ROS generator. GA inhibited the survival of A549 and CL1-0 lung cancer cells (Figure 2A,B). To prove that the inhibition of cell survival by GA is due to ROS, we treated the cells with the well-known antioxidant NAC. Surprisingly, 10 mM NAC treatment did not restore cell proliferation, but enhanced GA-induced growth inhibition in A549 cells (Figure 2C). Therefore, we examined the effects of different concentrations of NAC on the proliferation of A549 cells. The results showed that 2 mM NAC had no effect on the proliferation of A549 cells (Figure 3A), but NAC at concentrations between 4 and 10 mM dose-dependently inhibited cell proliferation (Figure 3A). Although 2 mM NAC had no effect on cell proliferation, it could enhance the growth inhibition induced by GA (Figure 3B).

### 2.2. The Effect of Cotreatment with NAC and GA on the Proliferation of Cells Expressing Different PERP-428 Variants

**Figure 4:**
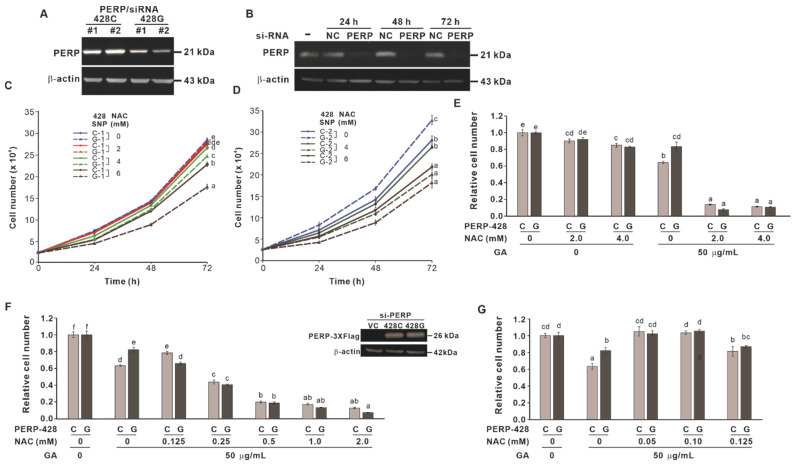
The effect of NAC and GA on the proliferation of CL1-0 cells expressing different *PERP*-*428* variants. (**A**) Expression of *PERP* protein in CL1-0/*PERP-428C* and CL1-0/*PERP-428G* stable clones. (**B**) Knockdown efficiency of si-*PERP* in A549 cells. si-NC: si-negative control. (**C**,**D**) NAC at 4 or 6 mM inhibited the proliferation of CL1-0/*PERP-428G*-expressing cells (dashed lines) much more significantly than that of CL1-0/*PERP-428C*-expressing cells (solid lines). One-way ANOVA and the Scheffé test were used to analyze the effect of NAC on the proliferation of cells expressing *PERP-428C* and *PERP-428G*; for (**C**): F(7,16) = 254.782, *p* = 1.9 × 10^−14^; for (**D**): F(5,12) = 68.427, *p* = 2.1 × 10^−8^. (**E**) NAC (2 mM and 4 mM) only slightly inhibited the proliferation of CL1-0 cells expressing *PERP-428C* or *PERP-428G*. However, 2 mM NAC and 50 μg/mL GA treatment completely inhibited cell proliferation. One-way ANOVA and the Scheffé test were used to analyze the effects of NAC and GA treatments on cells expressing *PERP-428C* and *PERP-428G*; F(11,24) = 813.994, *p* = 4.5 × 10^−28^. (**F**) NAC concentrations higher than 0.25 mM and GA treatment completely inhibited cell proliferation. One-way ANOVA and the Scheffé test were used to analyze the effects of NAC and GA treatments on cells expressing *PERP-428C* and *PERP-428G*; F(13,28) = 534.476, *p* = 5.6 × 10^−30^. (**G**) NAC concentrations below 0.1 mM eliminated GA-induced inhibition of proliferation. One-way ANOVA and the Scheffé test were used to analyze the effects of NAC and GA treatments on cells expressing *PERP-428C* and *PERP-428G*; F(9,20) = 44.410, *p* = 2.8 × 10^−11^. The letters in the figure indicate the homogeneous subsets, and data sharing the same letter are not significantly different as determined by the Scheffé test (*p* > 0.05).

Our previous studies have shown that GA-induced growth inhibition or cell death of lung cancer cells is dependent on the antioxidant capacity of the cells. CL1-0 cells expressing the *PERP-428G* genotype have higher antioxidant activity than cells expressing *PERP-428C*. In CL1-0 cells stably expressing *PERP-428C*, GA showed stronger inhibition of proliferation than that in cells expressing *PERP-428G* [12]. To investigate whether NAC differentially enhanced GA-induced growth inhibition in cells with different antioxidant activities, we first examined the effects of different concentrations of NAC on cells stably expressing *PERP-428* variants in which endogenous *PERP* was knocked down by si-RNA (3′UTR). Figure 4A shows the expression of *PERP-428C* and *PERP-428G* protein levels in stable clones. Since CL1-0 expresses low levels of *PERP*, we used A549 cells to demonstrate that si-*PERP* (3′UTR) effectively reduced *PERP* protein levels 24 h after transfection, and the effect lasted for 72 h (Figure 4B). The results showed that 2 mM NAC had no effect on the proliferation of the cells expressing *PERP-428C* and *PERP-428G*. However, when the concentration of NAC was increased to 4 or 6 mM, the growth-inhibitory effect of NAC on the *PERP-428G*-expressing cells was more significant than that on the cells expressing *PERP-428C* (Figure 4C). The same effect was observed in two other independent stable clones of each *PERP-428* variant (Figure 4D). To exclude possible artifacts generated by stable clones, transient transfection of *PERP-428C* and *PERP-428G* was performed in CL1-0 cells in which endogenous *PERP* was knocked down by si-*PERP* (3′UTR). Then, we combined GA (50 μg/mL) and different concentrations of NAC to treat CL1-0 cells that transiently expressed different *PERP-428* variants for 72 h. The results showed that the cells transiently expressing *PERP-428G* were more resistant to GA than the cells expressing *PERP-428C* (Figure 4E, compare lanes 7 and 8; Figure 4F,G, compare lanes 3 and 4). Although 2 and 4 mM NAC alone slightly inhibited cell proliferation, cotreatment with GA and 2 or 4 mM NAC greatly reduced the number of cells (Figure 4E). This may have been due to the induction of cell death because a large number of cells detached from the bottom of the well. When the NAC concentration with GA cotreatment was reduced to 0.25 mM, the proliferation of the two *PERP-428* variant-expressing cells was inhibited in a dose-dependent manner (Figure 4F). In contrast, when the NAC concentration was lower than 0.1 mM, the effect of GA-induced growth inhibition was abolished (Figure 4G). Therefore, we speculated that when the concentration of NAC in CL1-0/*PERP-428* SNPs cells is higher than 0.25 mM, it may act as a pro-oxidant and enhance GA-induced growth inhibition, and when the concentration of NAC is lower than 0.1 mM, it acts as an antioxidant and eliminates ROS induced by GA.

### 2.3. Cotreatment with NAC and GA Enhanced the Death of Cells Expressing PERP-428C

**Figure 5:**
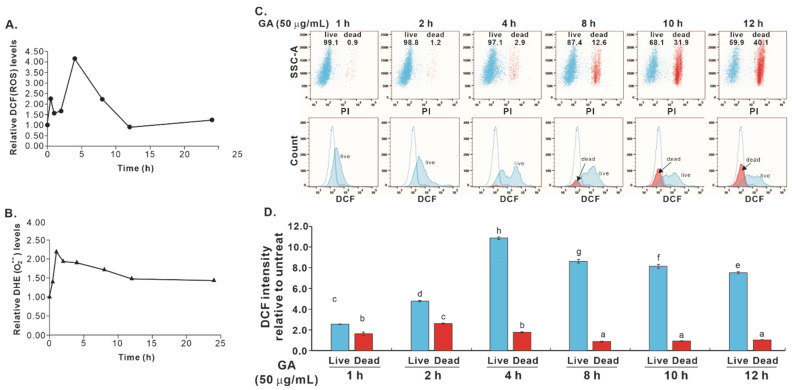
The effect of GA treatment on the intracellular ROS level of CL1-0 cells expressing *PERP-428C*. (**A**) ROS levels in CL1-0/*PERP-428C* cells within 24 h after GA treatment as determined by H_2_DCFDA. (**B**) DHE measures the level of superoxide anions in CL1-0/*PERP-428C* cells within 24 h after GA treatment. (**C**) Cell death (red in top panel) and ROS levels (bottom panel) in CL1-0/*PERP-428C* cells treated with GA within 12 h. Living cells (blue) had high ROS levels, but dead cells (red) had low ROS levels. (**D**) Quantitation of ROS levels in live and dead cells in (**C**). One-way ANOVA and the Scheffé test were used to analyze the ROS level of GA-treated cells at different time points; F(11,24) = 3847.946, *p* = 6.8 × 10^−10^. The letters in the figure indicate the homogeneous subsets, and data sharing the same letter are not significantly different as determined by the Scheffé test (*p* > 0.05).

Previous studies have shown that when H_2_O_2_ is present in the environment, NAC can become a pro-oxidant. Therefore, we investigated the changes in ROS levels in cells treated with GA or GA/NAC cotreatment. We previously observed that GA-induced *PERP-428C*-expressing cells began to die after 8 h of treatment, but *PERP-428G*-expressing cells did not die after 24 h of treatment [12]. Therefore, we first investigated the ROS level in the cells expressing *PERP-428C* at different time points after GA treatment. Within 30 min after GA treatment, the level of ROS in the cells expressing *PERP-428C* was increased, as shown by H_2_DCFDA treatment. It decreased 1–2 h after treatment; increased again 4 h post-treatment; and gradually decreased to the basal level 8–24 h post-treatment (Figure 5A). Similar fluctuations in ROS levels within 0.5 and 2 h after GA treatment were also observed in HeLa cells [15]. H_2_DCF is sensitive to H_2_O_2_, but less sensitive to superoxide anions. Therefore, we used DHE to detect the level of superoxide after GA treatment. The level of superoxide increased 1 h after GA treatment and then decreased slightly within 24 h (Figure 5B). Since CL1-0/*PERP-428C* cells began to die 8 h after GA treatment, we speculate that the decrease in ROS levels may be related to cell death. Therefore, we used PI to label dead cells and compared the ROS levels between dead and live cells for up to 12 h of GA treatment. The results showed that significant cell death was observed 8–12 h after GA treatment (Figure 5C, top panel; red represents PI-positive dead cells). The viable cells (blue) had high ROS levels, but the dead cells (red) had low ROS levels (Figure 5C, bottom panel). The quantitated ROS levels in live and dead cells are plotted in Figure 5D. At any time point after GA treatment, the dead cells were always associated with low ROS levels. It is not clear which occurs first, cell death or a loss of ROS.

**Figure 6:**
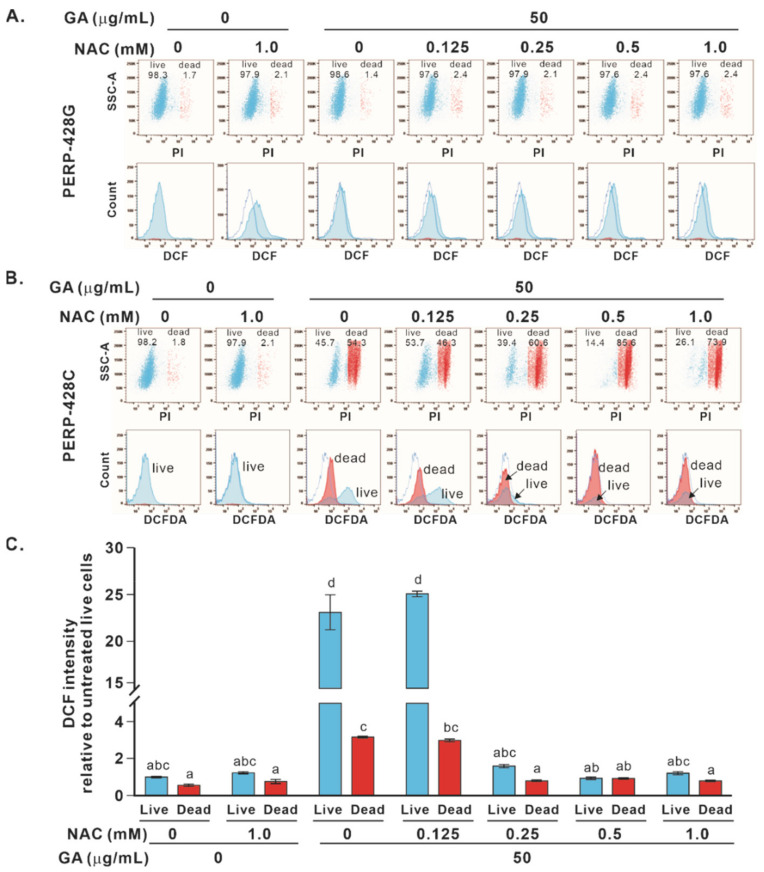
The effect of GA and NAC treatment on the level of intracellular ROS in CL1-0 cells expressing *PERP-428C* or *PERP-428G*. (**A**) The effects of NAC, GA and NAC/GA treatments on the death of cells expressing CL1-0/*PERP-428G* were determined by PI staining, and the effects on ROS levels were determined by H_2_DCFDA. After 24 h of treatment, NAC only slightly increased ROS without significant cell death. (**B**) For cells expressing CL1-0/*PERP-428C*, GA (50 μg/mL) treatment significantly increased ROS levels. Cotreatment with GA and higher NAC concentrations (0.25, 0.5 and 1.0 mM) enhanced cell death (red in top panel) and strongly reduced ROS levels (red in bottom panel). (**C**) Quantitation of ROS levels in (**B**). The living cells after GA treatment and GA and 0.125 mM NAC cotreatment had high ROS levels, but when the NAC concentrations were higher than 0.25 mM, the living cells had low ROS levels. One-way ANOVA and the Scheffé test were used to analyze the ROS between cells expressing *PERP-428C* and *PERP-428G* treated with GA and NAC; F(13,28) = 715.645, *p* = 1 × 10^−31^. The letters in the figure indicate the homogeneous subsets, and data sharing the same letter are not significantly different as determined by the Scheffé test (*p* > 0.05).

We next examined the changes in ROS levels in CL1-0/*PERP-428C* and *PERP-428G* cells treated with GA and NAC (0.125 mM to 1.0 mM) for 24 h. When CL1-0/*PERP-428G* cells were treated with GA and NAC (0.125 mM to 1.0 mM) for 24 h, the intracellular ROS increased only slightly (Figure 6A, bottom panel) and the cells were alive (Figure 6A, top panel). When CL1-0/*PERP428C* cells were treated with GA only for 24 h, 54.3% of the cells died (Figure 6B, red in top/third panel), with low ROS levels (Figure 6B, red in bottom/third panel); 45.7% of the cells were alive (Figure 6B, blue in top/third panel), with high ROS levels (Figure 6B, blue in bottom/third panel; Figure 6C). The combined treatments of GA and different concentrations of NAC increased the dead cell population (Figure 6B, top panel). Interestingly, the live cells in the 0.125 mM NAC and GA treatment group (PI-negative cells with intact membrane integrity) had high ROS levels, whereas the live cells in the 0.25 mM or higher NAC and GA cotreatment groups had low ROS levels (Figure 6B,C), suggesting that the loss of ROS precedes the loss of membrane integrity during cell death.

## 3. Discussion

GA (3,4,5-trihydroxybenzoic acid) is a natural plant phenol that can be used as an antioxidant at a concentration of 5 μg/mL [16], but at high concentrations, GA is an ROS producer [17]. GA induces cell death in cancer cells but has less cytotoxicity to fibroblasts and endothelial cells [18,19]. GA-induced death of lung cancer cells and HeLa cells is associated with increased ROS and glutathione (GSH) depletion [14,15]. Our previous studies have shown that the intracellular ROS level of CL1-0/*PERP-428C* cells is approximately two times higher than that of the CL1-0/*PERP-428G* cells. GA treatment significantly increased ROS and induced necrosis of CL1-0/*PERP-428C* cells in 8 h. In the CL1-0/*PERP-428G* cells that did not undergo GA-induced cell necrosis, GA treatment only slightly increased the ROS level. This phenomenon occurred because cells expressing *PERP-428G* have higher levels of antioxidant enzymes, catalase and glutathione reductase than cells expressing *PERP-428C* [12]. To show that GA-induced ROS were responsible for the death of cells expressing *PERP-428C*, we used NAC as an antioxidant. Unexpectedly, we observed that NAC concentrations higher than 0.25 mM dose-dependently enhanced GA-induced inhibition of proliferation in CL1-0 cells expressing *PERP-428* variants. However, when the NAC level was below 0.1 mM, GA-induced proliferation inhibition was eliminated in CL1-0 cells expressing *PERP-428C*.

**Figure 7:**
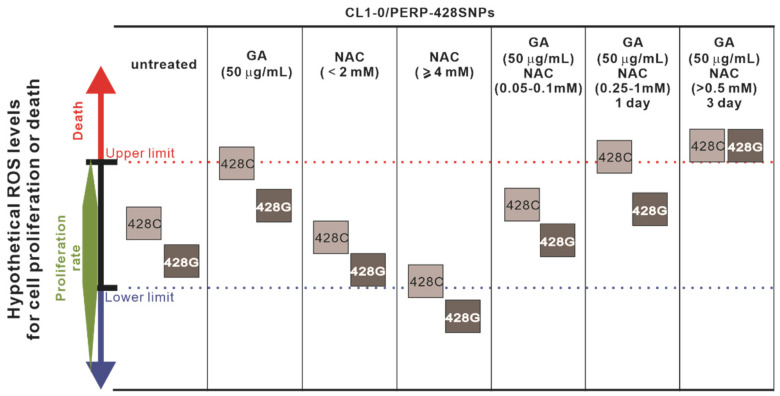
A diagram showing hypothetical ROS levels for cell proliferation or death after GA and/or NAC treatment. CL1-0 cells expressing *PERP-428C* or *PERP-428G* have different antioxidant capacities; therefore, they have different intracellular ROS levels (the first column). GA treatment increased ROS levels in *PERP-428C* cells to the upper limit of cell survival and induction of cell death (the 2nd column). NAC concentrations lower than 2 mM had no effect on cell proliferation, but NAC concentrations higher than 4 mM inhibited the proliferation of cells expressing *PERP-428G* more significantly than cells expressing *PERP-428C* (the 3rd and 4th columns). This effect may have been due to the antioxidant activity of NAC, which reduces the ROS levels of cells expressing *PERP-428G* to the lower limit of optimal cell proliferation. When the cells were cotreated with GA and NAC (0.05–0.10 mM), GA-induced cell death was eliminated in cells expressing *PERP-428C*, indicating that NAC acts as an antioxidant to reduce GA-induced ROS (the 5th column). However, in cells expressing *PERP-428C*, NAC concentrations higher than 0.25 mM enhanced GA-induced cell death (the 6th column). This phenomenon may be due to the high level of NAC in the H_2_O_2_ environment induced by GA becoming a significant pro-oxidant. Although GA cotreatment with NAC concentrations higher than 0.25 mM did not induce the death of *PERP-428G*-expressing cells in one day, most cells died on the third day after treatment (the 7th column).

Each cell type has a specific range of viable ROS levels that are required to maintain physiological functions, including proliferation, survival, differentiation, metabolism and angiogenesis. This is known as redox biology [20]. Oxidative stress involves high levels of ROS that cause damage to DNA, protein and lipids. Therefore, ROS levels that are too high are cytotoxic, and ROS levels that are too low are cytostatic [21]. We observed that treatment of A549 or CL1-0 lung cancer cells with NAC concentrations below 2 mM did not affect cell proliferation, but higher concentrations of NAC reduced cell proliferation. Interestingly, NAC showed greater dose-dependent inhibition of the proliferation of CL1-0 cells expressing *PERP-428G* than cells expressing *PERP-428C*. Our previous results indicate that cells expressing *PERP-428C* have higher intracellular ROS levels than cells expressing *PERP-428G*. NAC treatment may reduce the levels of ROS in cells expressing *PERP-428G*, exceeding the lower limit of ROS levels required for redox biology to support cell proliferation. Since the cells expressing *PERP-428C* have a higher basal ROS level, the reduction in ROS induced by a high concentration of NAC will not cause the intracellular ROS level to drop below the lower limit, so the final ROS level of cells expressing *PERP-428C* is still sufficient to support cell proliferation. Hypothetical intracellular ROS levels related to cell proliferation or cell death are illustrated in Figure 7. We found that 0.05–0.1 mM NAC abolished GA-induced proliferation inhibition, but when CL1-0/*PERP-428* SNPs cells were cotreated with 0.5 mM or higher NAC and 50 μg/mL GA, cell proliferation was completely inhibited, and many cells died. This phenomenon can be explained by NAC at a high concentration becoming a pro-oxidant in an environment containing GA. We and others have observed that GA produces H_2_O_2_, and H_2_O_2_ generates hydroxyl radicals through the Fenton reaction, thereby stimulating the pro-oxidant activity of NAC [8]. The free radicals generated by GA and NAC in three days will exceed the tolerance limit of CL1-0/*PERP-428* SNP cells, leading to inhibition of cell proliferation and cell death. In fact, CL1-0/*PERP-428G* cells were well tolerated in the cotreatment with 1mM NAC and GA. They survived well after 24 h of treatment but started to die after 48 h of treatment. On the other hand, in 0.25 mM or higher NAC and GA treatments, a high proportion of CL1-0/*PERP-428C* cells died within 24 h. Therefore, depending on the concentration of NAC, environment (presence of H_2_O_2_), and reaction time, NAC may become a pro-oxidant.

It has been shown that NAC enhances GA-induced cell death by increasing ROS levels and depleting GSH [13]. However, the authors found that the ROS levels increased within 24 h after treatment, which was not observed in our system. By 24 h, we found that a large number of CL1-0/*PERP-428C* cells had died, and ROS levels were very low. One possible explanation for the low ROS levels is that the DCF fluorescence diffused outside of the dead cells. We further observed that live cells (PI-positive cells) in the groups with GA and high levels of NAC cotreatment had reduced ROS levels before membrane integrity failed. We speculate that cotreatment with higher concentrations of NAC and GA may increase the level of ROS to a threshold, which greatly triggers the activity of antioxidant systems (such as GSH), thereby reducing the level of ROS in living cells. However, this may deplete GSH and eventually lead to cell death. Although we speculate that high concentrations of NAC in GA-treated cells act as a pro-oxidant, thereby enhancing GA-induced cell death, there may be other mechanisms by which NAC may induce GA-induced cell death. However, the mechanism of NAC’s pleiotropic function is still elusive. NAC enhances the growth inhibitory activity of EGCG in lung cancer cells by forming an EGCG-2′-NAC adduct, thereby stabilizing EGCG and enhancing EGCG-mediated cell death [22]. In addition, NAC (5 mM) has been shown to enhance imatinib-induced apoptosis of Bcr-Abl^+^ chronic myeloid leukemia cells by enhancing the production of endothelial nitric oxide (NO) [23].

This study revealed that NAC concentrations lower than 2 mM do not affect the proliferation of A549 and CL1-0 lung cancer cells. However, in A549 cells, 2 mM NAC significantly enhanced GA-induced growth inhibition. In CL1-0/*PERP-428C* cells, 0.25 mM NAC was sufficient to enhance GA-induced growth inhibition, and over 0.5 mM NAC and GA treatment induced cell death and completely inhibited cell proliferation. NAC has been proposed for cancer prevention or treatment [24,25,26,27] and as an adjuvant in treating many chronic diseases, such as liver and bowel diseases, metabolic syndrome, infectious disease, and neurodegenerative disorders [14,28]. In clinical trials, the oral administration dose of NAC ranges from 600 to 6000 mg/day, and the maximum plasma NAC concentration was observed to range from 0.012 to 0.123 mM [29,30,31]. Some clinical trials used intravenous NAC at doses of 12.5–25 mg/kg/day, resulting in a maximum NAC concentration range of 0.406–1.22 mM [32,33]. These clinical trials showed that NAC has no obvious side effects. However, a recent paper demonstrated that NAC promotes intestinal tumor progression in mice [33]. Thus, it is important to understand the characteristics of NAC in terms of antioxidant/pro-oxidant activity. Our research suggests that intracellular redox status may affect the biological action of NAC, shifting from antioxidant to pro-oxidant, which in turn impacts cellular functions and survival. This study provides information about the safe antioxidant dose of NAC in cells not attacked by H_2_O_2_ and the pro-oxidant dose of NAC in cells attacked by H_2_O_2_. This information may be valuable for the development of anti-lung cancer treatments using NAC and other polyphenols. In addition, for high-dose intravenous NAC, it may be important to consider the patient’s ROS level.

## 4. Materials and Methods

### 4.1. Cell Culture and PERP-428 SNP Constructs

A549 lung adenocarcinoma cells were obtained from the Bioresource Collection and Research Center (BCRC, Taiwan), and CL1-0 lung adenocarcinoma cells were kindly provided by P-C Yang (Department of Internal Medicine, National Taiwan University, Taipei, Taiwan) [34]. The *PERP-428* SNP is listed in the dbSNP (rs648802, NM_022121.5:c. 428C > G, NP_071404.2:p. Pro143Arg, P[CCT] > R[CGT]). *PERP-428C* and *PERP-428G* cDNA clones were generated by constructing polymerase chain reaction (PCR) fragments of cDNA derived from A549 (*PERP-428CG* heterozygous) mRNA into the PIRES-hr-GFP-1a plasmid and verified by DNA sequencing. CL1-0/*PERP-428C* and CL1-0/*PERP-428G* stable clones were established by transfecting *PERP* low-expressing CL1-0 cells with *PERP-428C*- or *PERP-428G*-expressing vector, and stable clones were selected with puromycin (2 μg/mL) [10].

### 4.2. Cell Proliferation Assay

The effect of NAC and/or GA on cell proliferation was assessed in A549, CL1-0, CL1-0/*PERP-428C* and CL1-0/*PERP-428G* stable clones or CL1-0 cells transiently transfected with *PERP-428C* and *PERP-428G*. CL1-0 expresses low levels of *PERP*, but it is homozygous for *PERP-428G/G*. To exclude the endogenous *PERP* effect, we knocked down endogenous *PERP* by transfecting CL1-0/*PERP-428C* or CL1-0/*PERP-428G* stable clones with si-*PERP* [3′ untranslated region (UTR); 5′-GCUUCAUGUUGACGAUA-3′] using Lipofectamine 2000 reagent (Invitrogen, Carlsbad, CA, USA). The cells were incubated at 37 °C in a 5% CO_2_ incubator for 24 h. For ectopic expression of *PERP-428C* or *PERP-428G*, 25 pmol si-*PERP* (3′UTR) and 2 μg plasmid DNA containing *PERP-428C* or *PERP-428G* were used to cotransfect CL1-0 cells using Lipofectamine reagent. After 24 h of incubation, the transfected cells were seeded in 12-well plates (3 × 10^4^ cells/well) and incubated for 16 h. The cells were treated with GA (50 μg/mL) and/or NAC (specified concentration). Every 24 h after treatment, the number of cells was determined by trypan blue dye exclusion in a hemocytometer.

### 4.3. ROS Detection and Propidium Iodide (PI) Staining

To examine the effects of GA and/or NAC on the intracellular ROS level and cell death of CL1-0/*PERP-428C* and CL1-0/*PERP-428G* cells, CL1-0 cells were transfected with 25 pmol si-*PERP* (3′UTR) and 2 μg *PERP-428C* or *PERP-428G* plasmid DNA using Lipofectamine and cultured at 37 °C for 24 h. Then, 1 × 10^5^ cells were seeded in 6-well plates and incubated at 37 °C for 16 h. The cells were then treated with GA (50 μg/mL) and/or NAC (0.125–1.0 mM) at the specified time points and incubated at 37 °C for the specified incubation time. At the end of incubation, the cells were stained with 10 μM H_2_DCFDA (2′,7′-dichlorodihydrofluorescein diacetate, Invitrogen, Thermo Fisher Scientific, Waltham, MA, USA) in the dark for 30 min. These cells were then washed with 1× PBS, trypsinized, and harvested with 500 μL 1× PBS. For PI staining, 5 μL of PI (BD Pharmingen, San Diego, CA, USA) was added to cells and incubated at room temperature in the dark for 15 min, and then, the samples were analyzed by flow cytometry.

### 4.4. DHE Staining

Dihydroethidium (DHE, Invitrogen, Thermo Fisher Scientific, Waltham, MA, USA) was used to determine the level of superoxide anions in CL1-0/*PERP-428C* cells treated with GA. CL1-0 cells were transfected with si-*PERP* (3′UTR) and plasmid DNA containing *PERP-428C*. Then, 1 × 10^5^ cells were seeded in 6-well plates for 16 h and treated with 50 μg/mL GA at different time points. After the specified incubation time, the cells were stained with 10 μM dihydroethidium in the dark for 30 min. The cells were then washed with 1× PBS, trypsinized, harvested with 1× PBS and analyzed by flow cytometry.

### 4.5. Statistical Analysis

Triplicate samples for two to three independent assays were used for each assay. A representative graph for each experiment is shown. For the ROS and superoxide assays, we pooled 4 to 5 repeated samples for flow cytometry to collect sufficient numbers of attached cells since CL1-0/*PERP-428C* cells start to die 8 h after GA treatment. Statistical analysis was performed using SPSS statistical software (version 14, SPSS, Inc., Chicago, IL, USA). Data are presented as mean ± SD values. One-way analysis of variance (ANOVA) followed by a post hoc Scheffé test for further multiple comparison testing was used to analyze the significance of differences among groups. Data between groups were compared using Student’s *t*-test. Differences with *p* < 0.05 were considered significant.

## Data Availability

The data presented in this study are available on request from the corresponding author.

## Sample Availability

Samples of the compounds are available from the authors.
